# Behavioral Health at School: Do Three Competences in Road Safety Education Impact the Protective Road Behaviors of Spanish Children?

**DOI:** 10.3390/ijerph17030935

**Published:** 2020-02-03

**Authors:** Francisco Alonso, Adela Gonzalez-Marin, Cristina Esteban, Sergio A. Useche

**Affiliations:** 1 DATS (Development and Advising in Traffic Safety) Research Group, INTRAS (Research Institute on Traffic and Road Safety), University of Valencia, 46022 València, Spain; cristina.esteban@uv.es (C.E.); sergio.useche@uv.es (S.A.U.); 2 Department of Economic and Legal Sciences, University Center of Defense (Spanish Air Force Academy), 30720 Santiago de la Ribera, Spain; adelaglez@cop.es

**Keywords:** education in road safety, RSE, children, behavioral health, protective road behaviors, road safety, traffic crashes

## Abstract

*Background*: Education in road safety (also known as Road Safety Education—RSE) constitutes, nowadays, an emergent approach for improving present and future road behaviors, aiming at taking action against the current, and concerning, state-of-affairs of traffic crashes, through a behavioral perspective. In the case of children, and despite their overrepresentation in traffic injury figures, RSE-based strategies for behavioral health in transportation remain a “new” approach, whose impact still needs to be empirically tested. *Objective*: The aim of this study is to assess the impact of three key road safety skills of the Positive Attitudes, Risk perception and Knowledge of norms (PARK) model, addressed in RSE-based interventions, on the safe road behavior of Spanish children. Methods: For this cross-sectional study, a representative sample of 1930 (50.4% males and 49.6% females) Spanish children attending primary school, with a mean age of 10.1 (SD = 1.6) years, was gathered from 70 educational centers across all Spanish regions, through a national study on RSE and road safety. *Results*: Road safety skills show a positive relationship with children’s self-reported safe behaviors on the road. However, the knowledge of traffic norms alone does not predict safe behaviors: it needs to be combined with risk perception and positive attitudes towards road safety. Furthermore, the degree of exposure to previous RSE interventions was shown to have an effect on the score obtained by children in each road safety skill; on the other hand, road misbehaviors observed in parents and peers had a negative impact on them. *Conclusion*: The outcomes of this study suggest that education in road safety is still a key process for the acquisition of safe habits, patterns and behaviors among young road users.

## 1. Introduction

Considering that in today’s society, and in the great majority of the cases, schooling involves the movement of a considerably large number of individuals, the educative system can be considered a sphere that is closely related to the transportation industry and its dynamics [1]. In this regard, health and safety outcomes related to schooling, especially whenever they are negative, represent a high burden for governments, in economic, productive and societal settings. Furthermore, the fact that traffic-related crashes constitute one of the main causes of mortality of children and adolescents worldwide suggests the need to develop studies and measures for reducing the road risks of this population; this can be achieved by strengthening the amount and quality of safe mobility information and the skills they are taught, especially considering that their most common trip is the commuting between home and school [2,3].

Although student transportation, especially in the case of children, is typically associated with “yellow school buses”, in most countries with a system different from the one employed in the United States students use different means of transport for daily commuting, some of them more organized and safer than others [3,4]. This implies, of course, a large set of risk factors for the health and welfare of children, and traffic crashes are, perhaps, the most relevant one from a global public health perspective [1,5]. Furthermore, approximately half of the fatal road crashes that take place worldwide involve road users that can be considered vulnerable, with children and aging adults being two vulnerable groups in terms of injuries and deaths derived from traffic accidents, especially in developing countries [1,6,7]. Nevertheless, much has been achieved in the case of other countries that, through the implementation of different measures, strategies and improvements, has reduced the morbimortality of young road users in recent years [8]. Considering this scenario, and the possibility of continuing to prevent crashes that involve young people, Road Safety Education (RSE) is an alternative form of reducing the number of injuries and deaths suffered by different road users, including children.

### 1.1. Road Safety Education: Value and the Shortcoming of the Evidence

Road Safety Education can be understood as the curriculum of educational actions on the topic of road safety and its related issues that are provided to the population in different scenarios, especially primary and secondary schools and social service agencies [9]. The value of Road Safety Education has been highlighted in recent decades as a manner of improving the behaviors, attitudes, knowledge and other psychosocial factors of people (especially among vulnerable groups of road users), in order to prevent road causalities through the strengthening of safety-related behaviors.

However, delivering Road Safety Education is not easy, cheap nor tautologically effective, and several shortcomings remain visible in RSE around the world.

First, it is worth mentioning that, to date, the effectiveness of Road Safety Education is relative: there are numerous successful and unsuccessful experiences around the world, and the first ones have been frequently linked to key factors such as the scope, quality, resources and systematicity of such interventions [10,11]. In other words, the issue of RSE effectiveness seems to be not about coverage—how many interventions regardless of their complexity and quality are performed—but about how often it is conceived as a systematic process.

Secondly, it is worth mentioning that, although this is the way they are commonly simplified, not all road safety activities (e.g., talks, events, mass-media campaigns) can be considered Road Safety Education interventions, since the latter ideally involves the need to develop a curriculum capable of methodically doing a follow-up and evaluating the results.

In addition, very few studies on Road Safety Education have evaluation criteria, e.g., behavioral performance and/or crashes suffered during the years that followed the intervention, a fact that limits the demonstrability of further results of this approach in improving the actual road safety of its beneficiaries. Thus, although there are studies suggesting that some potential long-lasting improvements may occur when good interventions are delivered, there is relatively little evidence to suggest that RSE contributes to the reduction of road risk in the mid- and long-term [12,13] or that it has a considerably small effect on the self-reported behavior of young people [14].

Finally, another issue frequently used for criticizing the effectiveness of RSE outcomes is the fact that, in several cases, many young people who behave unsafely are already aware that their behavior is unsafe [11], which implies the need to deliver not only information (knowledge of the norms and signals that may be necessary but not sufficient for crash prevention) but also of working hard on other key skills, such as risk perception and attitudes towards road safety [15,16]. In this regard, school emerges as the best scenario for holistically performing this task.

Studies performed in various countries support the assumption that prevention in road settings is a task that can be strengthened through schools [17], given, e.g., their primary role in education, their qualified personnel (teachers) and the availability of different resources (such as classrooms, computers, multi-media facilities, etc.) that are necessary to deliver Road Safety Education [18].

### 1.2. RSE for Children: The Same Topic but a Different Scope

Although it may sound redundant, the delivery of RSE cannot be the same for children and for other population segments. In the same way that age-based particularities within the explanation of traffic safety outcomes of different road users are relevant, the mechanisms and factors preventing accidents may be relatively specific for each age group. In this regard, recent research points towards the existence of differential needs in terms of education and training for children and young people, considering their psychosocial developmental stages, motivations and transportation patterns; these, in the case of children, involve the school environment and the commuting trips from/to school [8,9]. In fact, other relevant approaches support this assumption. The GDE Matrix (Goals for Driver Education) [19], initially aimed at training novice drivers, provides a framework used for defining educational goals and contents when training different road users under diverse approaches [20]. Although the GDE Matrix is used for developing RSE interventions in several population segments, it also considers the specific requirements of, e.g., different age groups, in order to strengthen the competencies and goals that are more pertinent for each one of them. In the case of children, and very similarly to what is observed in the positive attitudes, risk perception and rule knowledge (PARK) model, it usually focuses on improving the knowledge of road safety settings (e.g., norms, signals), the awareness on risk-increasing aspects and the self-assessment of children in their daily transportation tasks/situations.

Thus, the needs identified for earlier life stages seem to be different, and they appear highly related to the integration of Education for Road Safety (RSE) in curricular activities and school-based programs that consider specificities and particular factors such as children’s age, gender, personality, cognitive skills and social environment, primarily composed of e.g., parents, relatives, peers and other relevant stakeholders [7,21,22]. Actually, some studies addressing the practices related to the school-based RSE have found that, in countries such as the United States, Road Safety Education is much more developed in primary schools; here, the interventions are frequently directed by a single professor that is more involved in the activity development, which may strengthen the participation and the consistency of the interventions throughout time [10].

In addition, it is important to point out that it has been suggested that RSE should be imparted since the first years of schooling, and continued through primary and secondary school, in order to increase its effectiveness [11,23]. Although one of the greatest shortcomings of Education in Road Safety is that it focuses on the intra-personal features and skills of its beneficiaries and not on the actual modification or optimization of the objective road environment (e.g., infrastructural factors and transportation dynamics), it is worth saying that behavior constitutes, perhaps, the main predictor of road traffic causalities during the entire lifecycle. Therefore, strengthening road safety skills among children is, nowadays, considered a public health strategy for preventing potential further negative outcomes in transportation settings [22,24,25].

### 1.3. The PARK Model: Positive Attitudes, Risk Perception and Knowledge of Rules as Three Core Road Safety Skills

Interventions in Education for Road Safety address, worldwide, different skills and competences that may positively influence the road behavior of the population. However, as it has been previously mentioned, the available evidence on the ulterior impact of these interventions remains scarce to date; only few research experiences have tested the predictive value of road safety skills on the safe behaviors of, for instance, children and young people. In this regard, the existing evidence that uses the PARK model developed by Useche et al. [22], based on three key skills (i.e., positive attitudes towards road safety, risk perception and knowledge of traffic norms), suggests that safe road behaviors can be expected to be more numerous when children and adolescents have more positive attitudes towards road safety, a higher perception of road risks and a better knowledge of traffic norms and signaling [22,26].

Further studies performed in different countries have also associated risk perception in road safety issues with transportation patterns and safety outcomes [27,28,29], the positive attitudes towards road safety developed in young populations with their safety-related practices [16,30] and a better knowledge of norms with fewer risky behaviors [31], being that behavior is an often-used predictor for traffic crashes in different population and road-user groups [32].

In addition, bearing in mind previously documented RSE-related interventions that addressed similar road safety skills, the success of these actions seems to be related to their complexity, structure and systematic application over time. In other words, Education for Road Safety is more effective when there are consistent interventions on both socio-cognitive and behavioral issues (instead of isolated or single actions). This is especially true whenever the profile of their beneficiaries in terms of, e.g., age, gender and educational level are considered, in order to provide contents prone to be properly understood and applicable to a population’s daily road-related activities, actual behaviors and potentially involved stakeholders, such as their parents and peers [33].

### 1.4. Observational Learning also Plays a Role in Safety-Related Behavior

In the same way that the effectiveness of RSE-related programs has begun to show a positive impact on the safe behaviors of children and young people, there is a major behavioral contributor to children’s behavior that should not be omitted: children learn in an observational way, and their parents, figures of authority and peers play a significant role in the learning of safe patterns, habits and behaviors [34,35]. In other words, key processes such as observational and social learning may influence children’s road safety behaviors, either in a positive (strengthening their safe habits and preventing, for instance, traffic violations or risky decisions) or a negative way (especially those risky behaviors perceived in parents and peers, that can be easily imitated or assimilated as “non-problematic” by children) [26,35].

Typically, children may learn safe road habits from the behaviors observed in their daily life, even before taking up, e.g., the role of drivers in future stages of their life: some studies have shown how, for example, the driving behavior of parents, that is systematically observed by children, may modulate the way in which they will make decisions and/or assume risks as future drivers [25,26], especially during their young adulthood, which is a critical stage for their road safety. This is especially worrying when considering that young drivers between 18 and 26 years of age constitute a high-risk group with a considerable overrepresentation in accidents and fatalities worldwide [36]. In addition, the fact of perceiving very frequent road misbehaviors in the social environment can represent a latent constraint for the learning and assimilation of contents on road safety provided by the school or by campaigns on road safety [34,37,38]. This involves, of course, the road safety skills addressed in RSE interventions, and it implies the need to make the boundaries of school-based education even more extensive in children’s key scenarios, such as the parental and microsocial spheres [11,16].

### 1.5. Enhancing the Learning of Safe Road Behaviors in Children: The Role of School-Based RSE

Recent evidence shows how the strengthening and promotion of safe (or protective) road behaviors among young road users can be substantially reinforced by means of different interventions and strategies related to Education in Road Safety (RSE) [10,16,33]; this is especially true if we consider that, even though young users’ primary role on the road is that of pedestrians, they get progressively involved in driving tasks by using small (nonmotorized) vehicles such as bicycles and scooters. This represents, in most cases, a systematic transition towards the role of driver, which implies, in most countries, the necessity of attending road training and education programs provided by driving schools [13,36,39,40]. However, and especially in the case of low- and mid-income countries, this can be quite problematic: in fact, we need to keep in mind that the first contact with formation on road safety could take place when the individual is around 16–20 years of age, but these youngsters have often been frequent road users since their early childhood, and, thus, these skills are required to enhance their road safety during the entire lifecycle [13,40].

In this regard, the RSE paradigm acquires a crucial relevance for road safety, and it has been progressively adopted in different countries (including Spain), through its insertion in the educational sphere: this means that different interventions (e.g., signatures, events, conferences, visits to specialized centers, lessons on road safety in the classroom) have been taking place during the last two decades. In the particular case of Spain, RSE-related interventions follow the guidelines and materials [41] provided by the Directorate-General of Traffic (DGT, attached to the Ministry of Home Affairs), designed to facilitate and strengthen the standardization of Road Safety Education teaching in contexts such as the school one.

However, to date the evidence supporting the positive effects of Education for Road Safety on behavioral settings (i.e., having a “safer” behavior as a road user) is still scarce, and more scientific efforts are needed in order to support the role of RSE in the prevention of human-based traffic casualties. This was, indeed, the core reason behind the performance of this study: further interventions and improvements in Road Safety Education (RSE) should consider empirical evidences that test its actual impact on safe behavior, so that they can be strengthened and, also, systematically improved.

### 1.6. Objectives and Hypotheses

The core aim of this study was to assess the effect of demographic variables and of three key road safety skills (PARK; attitudes-risk perception-knowledge), addressed in RSE-based interventions, on the safe (or protective) road behaviors of Spanish children.

For this study, it was hypothesized that, in addition to demographic variables such as age and behaviors observed in peers and parents, the three addressed road safety skills would have a positive and significant effect on the self-reported protective road behavior of children.

## 2. Materials and Methods 

### 2.1. Sample

This cross-sectional study involved data collected from a sample of 1930 (973—50.4% male and 957—49.6% female) Spanish children attending primary school, ranging between 6 and 14 years of age, with a mean age of *M* = 10.10 (*SD* = 1.6) years.

### 2.2. Study Design and Procedure

Participants were invited to partake in the study through a nonprobabilistic (convenience) sampling method: as this study was part of a national-coverage survey on Road Safety Education, educational centers were systematically involved in the data collection; this sampling method is commonly used for this type of study, which is focused on specific populations, and it is based on the accessibility to the population and on their willingness to get (or not) involved in the research process. The (paper-based) questionnaires were completed in the classroom. The global response rate (completed and totally answered questionnaires) was approximately 96%, from a total of approximately 2000 primary school students initially asked to participate.

The sample size was calculated using the Raosoft^®^ sample size calculator (Raosoft Inc., Seattle, WA, USA), based on the total population and on the estimated sample needed to fulfill the basic parameters. The minimum sample size calculated was approximately 670 subjects, assuming a confidence level of 99% and a maximum margin of error of 5%. The study sample was determined to be representative of the Spanish population considering not only its large size but also its concordance with the characteristics of the population and its geographical coverage. In this regard: (a) this study retrieved data from 70 different educational centers across all 17 regions or autonomous communities of Spain; (b) 99.6% of participants were between 6 and 12 years old, that is the age interval regulated by the Spanish educational system; and (c) the sample kept the sex-based proportionality of subjects enrolled in primary schools for 2018, that was 51% males and 49% females [42].

As for procedural considerations, it is worth remarking that all the data were obtained from educational centers that had previously and officially agreed to partake in the research project, and questionnaires were always applied with the prior authorization and supervision of the educational staff (professors, coordinators and delegates) included in the pedagogical scheme of the center. Considering the average age of participants and the need for guaranteeing an adequate understanding of all the items included in the questionnaire, (a) the research tools were priory assessed through a “pilot” application, from which every hard-to-understand question or uncommon word was amended; (b) an associate of the study staff was permanently available to solve any doubts or questions that could arise during the completion of the questionnaire; (c) participants were informed that this study did not represent an academic test or evaluation and, thus, there were no right or wrong answers; and (d) especially considering that it dealt with underaged subjects, the survey was designed to preserve the anonymity and integrity of participants, highlighting the existing data protection regulations and the exclusively scientific purpose behind the data collection. This also contributed to minimizing the possibility of finding biases related to social desirability and/or acquiescent response styles. Finally, it is worth mentioning that a previous permission signed by the educational centers and their parents’ associations were obtained prior to the data collection phase. All the children involved in the research were initially informed about the importance of answering honestly to all the questions and about the importance of the topic and the inexistence of potential rewards (whether monetary or nonmonetary) as a consequence of their partaking in the study.

### 2.3. Instruments

For this research, a paper-based questionnaire composed of four sections was made up. The average time required for answering the survey in this sample of children was approximately 15 minutes. The questionnaire was structured as follows:

The first section inquired for a short set of basic demographic data (i.e., age, sex, city of residence, current school grade), in order to allow for the characterization of respondents.

A second part assessed three core factors related to Road Safety Education (RSE) at school, asking—in a scale ranging from 0 to 5—for the frequency/periodicity (0 = never to 5 = at least once a month), duration/intensity (0 = less than one hour to 5 = various days of the week) and complexity (0 = it was quite simple, and it did not use any resources to strengthen the experience to 5 = it implied several resources and contents to learn) of the school-based RSE-related activities in which children had been involved in the past, in order to determine both the involvement in and the nature of these actions.

A third section was designed to assess the three key road safety skills that the evidence has shown to be generally strengthened by RSE interventions, in accordance with the PARK model: positive attitudes, risk perception and knowledge of traffic rules [22]. We used, respectively, the following instruments suggested by the model [26]:

Positive attitudes (PA) towards road safety were measured by means of a 6-item scale (α = 0.74) that showed six statements related to safe/unsafe attitudes of road users (example item: “even if using the seatbelt were not mandatory, I would still wear it”). Secondly, road risk perception (R) was assessed through a 12-item scale (α = 0.63) that listed risky road situations, in order to determine how much risk the children perceived in each one of them (example item: “using the cellphone while walking or driving”). This was done through a Likert scale ranging from 0 to 2, where 0 = No risk perceived (“It does not constitute any risk for me; I do not think this could lead to a crash”); 1 = Mid risk perceived (“Although it might not necessarily cause a crash, it is true that this could put me in danger”); and 2 = High risk perceived (“It is definitely very dangerous and would surely put me at risk of suffering a crash”). The rule knowledge (K), i.e., knowledge of traffic rules and signals (α = 0.72) and ability to identify them, were assessed through a 12-item scale; it offered 12 statements on general road safety regulations to be labeled as false (F) or true (T), with the aim of assessing the children’s knowledge (example item: “rear seat passengers in a vehicle do not need to wear seatbelts”). The full set of items that composed the scales for assessing the PARK model skills is presented in Table 1.

Finally, the last section of the questionnaire addressed two factors:

Observed misbehaviors were measured through a three-level (0 = Never; 1 = Sometimes; 2 = Very Often) frequency-based Likert scale (α = 0.74), to assess how often the children observed risky behaviors in, e.g., parents and peers (6 items for parents and 6 for peers (example item: “how often do your parents drive after drinking alcohol?”). Finally, the self-rated safe (or protective) road behaviors were measured through a 6-item questionnaire (α = 0.79), used for assessing the extent to which children perform safety-related behaviors (example item: “when I am about to cross and the pedestrian traffic light has started blinking, I prefer not to cross, waiting for the next green”) that may contribute to preventing road crashes. 

### 2.4. Statistical Analysis (Data Processing)

After a careful data curation, study variables were calculated: in the case of demographics, the data were rigorously coded and labelled; as for sub-scales (RSE-related skills), factors were scored using the guidelines of each instrument. Although only some questionnaires contained missing values in some of the questions, and the sample was considerably large, missing data were transformed through regression coefficient-based data imputation (procedure available in AMOS 26.0 software; Armonk, NY, USA), in order to respect the distribution trends of the data and their descriptive and dispersion parameters, such as the mean (M) and the standard deviation (SD).

Furthermore, a set of bivariate correlational analyses (Spearman’s *rho* or *r_s_*) was carried out, with the aim of establishing relationships between pairs of the study variables. This analysis was chosen keeping in mind its robustness over Pearson’s association coefficients (*r*) when scales involved ordinal data, such as the case of many Likert-based questionnaires [43]. The effect of the independent factors on the dependent variable (safe road behaviors) was assessed through path analysis (Structural Equation Modelling, or SEM) using maximum likelihood estimations. The following statistical parameters were considered for addressing the significance of the paths under different confidence levels: *p* < 0.05, *p* < 0.01 and *p* < 0.001. All the statistical procedures were performed using ©IBM SPSS (Statistical Package for Social Sciences), version 26.0 (Armonk, NY, USA, 2019), and ©IBM SPSS AMOS, version 26.0, especially for conducting structural (SEM) analyses, that require a specialized software.

### 2.5. Ethics

This study was framed within the macro-project “Sustainable Mobility: Behavior, Lifestyle, Health and Safety of Non-Motorized Road Users”, and approved by the Ethics Committee of the University of Valencia with the IRB H1535548125595, thus certifying that the research subject to analysis responded to the general ethical principles, currently relevant to research in Social Sciences.

## 3. Results

### 3.1. Bivariate Correlation Analysis

The Spearman’s (*r_s_*) correlation analysis showed significant associations (most of them moderate) between the different variables (when pairs of study variables are crossed), as shown in Table 2. The most relevant correlations, essentially those related to RSE and the three measured road safety skills (risk perception-attitudes-knowledge) were theoretically coherent and are described as follows:

Firstly, received RSE was positively and significantly associated with risk perception (*r_s_* = 0.060 *), positive attitudes towards road safety (*r_s_* = 0.055 *), traffic rule knowledge (*r_s_* = 0.061 *), and with the self-reported safe behaviors performed by children (*r_s_* = 0.095 **). Risk perception showed a significant and positive correlation to the other two road safety skills (attitudes—*r_s_* = 0.261 ** and rule knowledge—*r_s_* = 0.128 **) and to safe behaviors (*r_s_* = 0.162 **), but it was negatively linked to observed misbehaviors (*r_s_* = *−*0.094 **). Positive attitudes were also correlated to rule knowledge (*r_s_* = 0.116 **) and safe behaviors (*r_s_* = 0.215 **) and negatively related to observed misbehaviors (*r_s_* = *−*0.188 **). Finally, rule knowledge was positively associated with safe behaviors (*r_s_* = 0.072 **) and observed misbehaviors (*r_s_* = 0.052 **), as shown in Table 2.

### 3.2. Effect of Study Variables on Safe Road Behaviors

Based on the theoretical assumptions described in the background, the effect of Education for Road Safety (RSE) interventions on safe road behaviors was assessed through Structural Equation Modeling (SEM). The structural analyses, based on the directionality and relationships of the hypothesized paths, were fitted to the data retrieved from the full sample of n = 1903 Spanish children.

**Figure 1:**
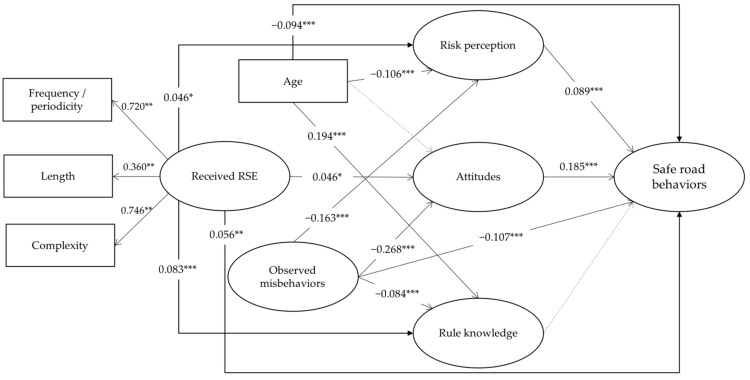
Path structural model presenting standardized path coefficients for self-reported Safe (protective) Road Behaviors: * *p* < 0.05; ** *p* < 0.01; *** *p* < 0.001. Solid lines (arrows) represent significant paths and intermittent lines represent nonsignificant ones.

As suggested by the specialized literature in the field [44,45], structural modeling requires a baseline (unconstrained) model, that in this case did not fit the data very well (*x*^2^_(6)_ = 159.225, *p* < 0.001; Normed Fit Index (NFI) = 0.764; Comparative Fit Index (CFI) = 0.763; Root Mean Square Error of Approximation (RMSEA) = 0.115 − CI_95%_ [0.100–0.131]). It needed to be corrected by considering the relationships and intercorrelations between the different study variables. Consequently, a short set of modifications was performed. Firstly, nonsignificant and very low paths in the baseline model were set to zero. Furthermore, Modification Indexes pointing out relationships between the independent variables and the protective (safe) road behaviors were considered to constrain the initial model. These changes made the model even more parsimonious, and the model fit that resulted was adequate. The resulting Structural Equation Model (constrained SEM) was better fitted and more parsimonious (*x*^2^_(4)_ = 29.058, *p* < 0.001; NFI = 0.957; CFI = 0.961; RMSEA = 0.057 − CI_95%_ [0.039–0.058], all of them were suitable and suggested an adequate model fit [45]. The full set of paths contained in the retained model are shown in Table 3, in which the arrows indicate the directional relationship between the variables (exogenous/independent → endogenous/dependent) included in the model. Furthermore, the model is graphically presented in Figure 1.

The results of the SEM model, in which continuous arrows indicate significant paths (see Figure 1), suggest that:

1. Age has a significant effect on rule knowledge (*β* = 0.194 ***) and its effect on risk perception is negative (*β* = −0.106 ***); it also had a negative relationship with the performance of safe (protective) road behaviors (*β* = −0.094 ***) among children.
2. Received Road Safety Education (RSE) has a positive and significant effect on the three addressed road safety skills: risk perception (*β* = 0.046 *), attitudes towards road safety (*β* = 0.046 *) and traffic rule knowledge (*β* = 0.083 ***). In addition, received RSE has a significant effect on the self-reported road protective behaviors (*β* = 0.056 **).
3. Just as the received RSE, observed misbehaviors in parents and peers also have a significant (but negative) effect on road safety skills: risk perception (*β* = −0.163 ***), attitudes towards road safety (*β* = −0.268 ***) and rule knowledge (*β* = −0.084***), as well as a negative and significant effect on the performance of safe road behaviors (*β* = −0.107 ***).
4. Two out of the three road safety skills (i.e., risk perception—*β* = 0.089 ***; *R*^2^= 0.061, and positive attitudes towards road safety—*β* = 0.185 ***; *R*^2^ = 0.082) have a significant and positive (but considerably small in terms of magnitude and explained variance) effect on the safe road behaviors performed by children, also exerting a statistical mediation (partial) between age, RSE, observed misbehaviors and the dependent variable. Rule knowledge explains less than 6% of the variance (*R*^2^ = 0.053) and does not exert a significant effect on the self-reported safe behaviors.

## 4. Discussion

The purpose of this study was to assess the effect of demographic variables and of three key road safety skills (PARK; attitudes- risk perception-knowledge), addressed in RSE-based interventions, on the safe (or protective) road behaviors of Spanish children. Overall, the findings of this research support the assumption that these road safety skills, that are the most generally addressed by different Education for Road Safety approaches, have a positive impact on the children’s self-rated safety behaviors on the road. In this regard, there are several particular findings that are worth discussing in the light of the theoretical roots presented in the introduction and in other previous research addressing similar objectives and variables in the field.

First of all, it is worth acknowledging that this study covered three skills that, although they potentially may not be covered by Road Safety Education-based programs and/or interventions, do address the standards followed by most of these actions in the Spanish context. In addition, we should consider the effect of key issues such as the frequency, length and complexity with which they are delivered to the participants. In this regard, the relationship between these three issues on the road safety skills composing the PARK model was positive and significant in all three cases, supporting what is already stated in different studies which claim that the systematic character of the interventions in RSE may explain a better acquisition of these road safety skills [10,13,26]. Furthermore, in addition to the coherence of the directionality of the relationships found among these variables, there are significant correlations and slightly greater effects if the RSE-related actions display a higher frequency, duration and number of contents addressed, as previously suggested in similar studies [26]. Nevertheless, it should be considered that, although the observed correlations are statistically significant (which could be due to the use of a large sample size), the magnitude of some of the correlations remains relatively small (low to moderate), thus suggesting that their use can be avoided [46] and their interpretation should be, at least, careful. In addition, and regardless of the technical strategies used for avoiding, e.g., social desirability and acquiescence, the self-reported nature of the data does not guarantee their inexistence, especially since we are dealing with self-rated behaviors that may be, for instance, differently rated by proxies, such as parents and peers. Further information in this regard can be found in the limitations of the study.

In regard to the three competencies (or skills) composing the PARK model that were addressed in this study, age seems to play a relevant role in the explanation of the scores obtained by participants in terms of risk perception (that is higher in lower age segments) and knowledge of traffic norms; even though risk perception and knowledge of traffic norms seem to get better with age, they compose only a conceptual sphere that should be accompanied by a higher risk perception and more favorable attitudes to explain safe behaviors on the road. Among the three road safety skills measured in this study, rule knowledge was the only one not exerting a direct effect on the self-rated protective behaviors of children. A similar finding was documented in the case of Spanish male adolescents by Useche et al. [22], suggesting that behavioral competences a) jointly interact in the explanation of both risky and safe behaviors, and b) can also be more influenced by risk perception and attitudinal settings than by mere conceptual settings that need further processes (e.g., motivation, attitudes) to translate into action. In addition, Zeedyk et al. [47] found a similar outcome, thus supporting the theory that an increased knowledge on road-related normative does not necessarily imply behavioral improvements; this is especially true in the absence of complementary skills aimed at enhancing the decision-making of children in the road environment.

Another relevant variable measured in this research was the frequency with which children reported the road misbehaviors they observed in parents and peers, a variable which has a negative effect on all three road safety skills. This happens despite the fact that the variable is independent from the exposure to RSE-related interventions, which makes theoretical sense in the glance of the empirical literature dealing with observational learning in children [26,47,48]. However, the observed road misbehaviors presented a significant (and detrimental) effect on the learning of these three competences and, also, a direct and negative effect on the rate of safe road behaviors performed by children. In practical terms, it is noticeable in some research how this factor could constitute a latent constraint to the effectiveness of interventions related to road safety, especially when considering that school-based strategies do not constitute the only source of learning of safe behaviors, patterns and habits among children [21,27,47]. Although not addressed in this study, features related to the microsocial environment are also a relevant sphere that should be taken into account in order to formulate interventions that might potentially involve stakeholders who are external to the school environment, such as parents, friends and relatives, as a manner of holistically addressing this problematic issue [48,49].

Back to the outcomes of the present research, the results of the structural analysis also suggest that observational learning and parental influences have an effect on the road safety behavior of children. In this regard, it is worth summarizing some relevant findings contained in the literature, that even suggest this effect could be observed in further stages of life: cautious adult road users have been found to be more prone to have more cautious kids [50]; especially in the case of road misbehaviors, hazardous road behaviors shown by parents are usually emulated by their children in the future [51]. In this sense, and according to the findings (significant correlations) of the study that were related to observed misbehaviors, it can be concluded that the behavior of parents and peers does indeed influence the children’s attitudes, the learning of road safety norms and their perception of road risks, potentially affecting their behavioral outcomes, as well as (we hypothesize) their present and future road safety: this is a key finding of this study [52,53].

Another strength of this research was the use of structural models: this process, that made it possible to build up an explanatory model with good fit and coherent/significant paths, in accordance to both the theory and the hypothesized outcomes, has allowed us to conclude that safe behaviors are significantly influenced by three core skills that can be strengthened in the school environment, as it is also described in previous studies [26,54]. In this regard, and in consideration of both the current findings and what is mentioned in other previous studies, this research advocates for the importance of school-based RSE supported by quality budgets, systematicity and orientation towards key behavioral competences or *skills*—positive effects on behavior of RSE that have been observed when good practices are adopted [10,11]—as a way of enhancing its effectiveness and long-lasting impact on the present and future road behavior of children.

Moreover, the results of this experience highlight the explanatory link between latent factors and actual behaviors reported by children, and this has been a common constraint to the performance of further predictions in merely descriptive studies, which usually employ smaller or excessively local samples. Nevertheless, it is worth remarking two potential shortcomings related (respectively) to the structural analysis and the contents of the instruments we used: first that—although significant—the Standardized Path Coefficients (SPCs) remain considerably low in magnitude (which does not invalidate the results but suggests their careful interpretation) and, second, that for this study we did not consider in depth the emergence of new factors that could potentially get involved in the road safety dynamics of children, and which may also threaten or compromise their behavioral performance, such as cellphones and other information and communication technologies [55,56]. It is noticeable how the use of technological devices has been progressively becoming more and more frequent in the young populations, as part of other research and interventions in the field of Education for Road Safety, and should be therefore considered in further studies addressing a similar research problem. In other words, and according to the short predictive value shown by the knowledge of rules and traffic norms alone (that is, perhaps, the most commonly addressed skill in these interventions), standardized contents on RSE should consider current trends and new factors involved in the transportation dynamics of children; especially if they share the idea that RSE inputs introduced during the first stages of life can be translated into better behavioral outcomes, thus maximizing the efficacy and efficiency of RSE interventions [10,26,49].

Finally, two key facts must be remarked: first, that more research on the quality, structure and effectiveness of behavioral interventions on road safety is needed in order to determine the most effective (and ineffective) ways of improving road safety behaviors [57]; and, second, the need to not only deliver more RSE interventions but also to improve the mechanisms used for their design (contextual factors seem to be highly relevant), evaluating their impact on the actual behavior and road safety of children in the mid- and long-term. This is something that—although expensive and methodologically difficult—could contribute to demonstrating the permanence of the information provided by the Road Safety Education and could highlight any improvable shortcomings in its approach, design and deliverance. This requires, naturally, several efforts towards the improvement of the articulation amongst the educational system and other spheres of life that may strengthen this task, such as micro-, macro-social, primary healthcare and policymaking systems.

### Limitations of This Study and Further Research

This study used a representative sample, whose size was considerably large; the study also covered all regions (autonomous communities) of Spain and kept a gender-based parity among participants. Statistical parameters were, overall, achieved, and the results show a high consistency with other theoretical and empirical reference sources. However, it is worth acknowledging some factors that could potentially bias the outcomes of this and other similar studies.

First, the data analyzed in this research were based on self-reports, thus enabling the existence of common method biases that could influence the responses provided by our participants. In this regard, young populations have shown to present more desirable response trends than, e.g., adults, when, for instance, they feel they are being evaluated [58,59]. Although we have previously mentioned that a clear description of both the objectives and the technical considerations followed by this study were presented to participants, this does not necessarily suppress the social desirability or acquiescence that they may have experienced. In addition, other studies using self-reported data on road behaviors performed by young people have pointed out the need to consider the awareness of subjects of their own road behaviors as a potential source of bias [14]. Furthermore, the information on behavioral settings may substantially differ from the data potentially provided by proxies/third parties or observational measures, as observed in previous studies dealing with children’s behavior in other spheres [60].

Second, our data collection was performed in the classroom, a relevant contextual factor that could have implied a certain predisposition of the participants to deliver “positive” or “desirable” responses in order to please the research staff. This can happen even in spite of the researchers’ remarks on the “non-existence of wrong or right responses” which was also explicitly written in the questionnaire.

Third, and although consistent correlations were found for risk perception, this variable reported a relatively low Cronbach’s Alpha (0.63). This could be due to the relatively small number of items on the scale (a choice that was made considering the total length of the survey) that included several other scales and considered that cause maturation/fatigue or nonresponse bias could be caused in the case of using longer scales for the final questionnaire. Additionally, other available questionnaires on young users’ road behavior, such as the Adolescent Road user Behaviour Questionnaire (ARBQ) [61] may prove useful to perform further measurements in this regard.

Finally, and although the instruments used in this research had been previously (and successfully) applied to similar populations in comparable age ranges, under the consideration that questionnaires applicable to children had to be as brief and succinct as possible [59,62], we did not assess the literacy and vocabulary-related abilities of our participants; the questionnaire was administrated regardless of their current academic year/level. However, in this regard, it is worth highlighting that all possible doubts and questions were immediately responded to by a member of the research staff, always present in the classroom at the moment of data collection.

## 5. Conclusions

The results of this study support the relationship among the three skills related to RSE (although not exclusively attainable through it) that compose the PARK model, i.e., positive attitudes towards road safety, risk perception and rule knowledge, and the safe road behaviors of Spanish children. The scores in these three skills are also positively influenced by the frequency, length and complexity of RSE-related activities. Further, positive attitudes towards road safety and risk perception have shown a significant effect on the self-reported safe behavior.

This study endorses the value of Road Safety Education for the development of behavioral skills to strengthen the prevention of human factor-related traffic crashes involving children and young people, under assumptions of well-designed programs, systematic interventions, continuous evaluation and improvement approaches.

## Figures and Tables

**Table 1:** Composition of the scales measuring the three skills of the positive attitudes, risk perception and rule knowledge (PARK) model.

Composing Items/Tasks	Scale	Scoring Guidelines	Mean Score	Std. Deviation
**PA (Positive Attitudes Towards Road Safety)**				
Even if it wasn’t mandatory, I would fasten my seat belt	Dichotomous; 0 = No, 1 = Yes	Summing all the values after recoding the negative items.	4.69	1.237
The use of the helmet should be voluntary, only for those people who want to use it				
Traffic signs and regulations only serve to give us fines ^(−)^				
People who do not comply with traffic norms can be fined				
It is boring to have to follow all road safety regulations ^(−)^				
When I travel by car, it seems good to me to overtake others as if it were a race				
**R (Risk Perception)**				
A person drives after having a couple of beers	Likert scale ranging from 0 = no risk perceived, to 2 = high risk perceived	Summing all the item values	17.32	2.48
Going as passenger of a driver who has drank alcohol				
A person drives when it is raining heavily				
Using the cellphone while driving				
Using the cellphone while walking				
A cyclist does not wear a helmet				
Traveling in a car while being in bad mechanical condition				
Assuming more risks because the road is in good condition				
Wearing a seat belt that is incorrectly adjusted				
Not using the seat belt because the car has an airbag				
Wearing a badly fitting helmet when riding a bike or skateboard				
Driving several hours in a row				
**K (Knowledge of Traffic Norms and Signals)**				
Traffic norms (written statements):	Dichotomous (True/False). Right answers are coded with 1, and wrong answers with 0	Summing all the right answers (1) to obtain a global score in the variable "knowledge of traffic norms and signals), that can also be separately summed, if needed.	9.522	1.214
In a crosswalk, I can cross without looking since the pedestrian always has preference				
I should always wear a helmet when riding a bicycle				
Rear-seat passengers in a vehicle do not need to wear a seat belt				
From the age of 12, I can go as a co-pilot in the front seat				
The maximum blood alcohol limit allowed to drive a motorcycle is 0.5 g/L.				
A driver waiting at a traffic light can answer a phone call				
Traffic signals (presented in slides by the tester):				
There is a pedestrian step				
Road reserved for pedestrian				
No overtaking				
Mandatory stop				
Wrong way				
Bike lane				

Notes: ^(−)^ Negative item.

**Table 2:** Bivariate (Spearman) correlations between study variables.

Study Variable		1	2	3	4	5	6
1	Age (years)	--					
2	Received RSE	−0.106 **	--				
3	Observed misbehaviors	0.106 **	−0.048	--			
4	Risk perception	−0.112 **	0.060 *	−0.094 **	--		
5	Positive attitudes	0.049 *	0.055 *	−0.188 **	0.261 **	--	
6	Rule knowledge	0.175 **	0.061 *	0.052 *	0.128 **	0.116 **	--
7	Safe behaviors	−0.106 **	0.095 **	−0.116 **	0.162 **	0.215 **	0.072 **

Notes: ** Correlation is significant at 0.01 level (2-tailed). * Correlation is significant at 0.05 level (2-tailed).

**Table 3:** Structural Equation Model (SEM) for predicting safe (protective) behaviors.

SEM Paths Composing the Retained Model			S.P.C. ^1^	S.E. ^2^	C.R. ^3^	*p* ^4^	Sig.
Received RSE	→	Rule knowledge	0.083	0.044	3.746	<0.001	***
Received RSE	→	Positive Attitudes	0.046	0.044	2.098	0.036	*
Received RSE	→	Risk perception	0.046	0.090	2.070	0.038	*
Age	→	Risk perception	−0.106	0.035	−4.730	<0.001	***
Age	→	Positive Attitudes	0.037	0.017	1.696	0.090	N/S
Age	→	Rule knowledge	0.194	0.017	8.755	<0.001	***
Observed Misbehaviors	→	Risk Perception	−0.163	0.057	−7.132	<0.001	***
Observed Misbehaviors	→	Positive Attitudes	−0.268	0.028	−11.92	<0.001	***
Observed Misbehaviors	→	Rule Knowledge	−0.084	0.028	−3.686	<0.001	***
Positive Attitudes	→	Safe Behaviors	0.185	0.021	7.955	<0.001	***
Risk perception	→	Safe Behaviors	0.089	0.010	3.909	<0.001	***
Rule knowledge	→	Safe Behaviors	0.035	0.021	1.543	0.123	N/S
Received RSE	→	Safe Behaviors	0.056	0.040	2.580	0.010	**
Age	→	Safe Behaviors	−0.094	0.016	−4.203	<0.001	***
Observed misbehaviors	→	Safe Behaviors	−0.107	0.026	−4.585	<0.001	***

Notes: ^1^ S.P.C. = Standardized Path Coefficients (can be interpreted as linear regression weights). ^2^ S.E. = Standard Error. ^3^ C.R. = Critical Ratio. ^4^ *p*-values. N/S = Non-significant path. *** = Significant at level 0.001. ** = Significant at level 0.01. * = Significant at level 0.05.
